# Supplementary material for: COZOID: contact zone identifier for visual analysis of protein-protein interactions
Source: BMC Bioinformatics. 2018 Apr 6;19:125. doi: 10.1186/s12859-018-2113-6 (PMC5889581; doi:10.1186/s12859-018-2113-6)
Supplement: Supplementary file 2 — Software build. Executable binary file of the software tool. (ZIP 118,784 kb) [file 12859_2018_2113_MOESM2_ESM.zip › cozoid/platform/docs/testng-6.5.1-javadoc.zip!/serialized-form.html]

Serialized Form (TestNG)


---


|  |  |  |
| --- | --- | --- |
| **Overview** \| Package \| Class \| **Tree** \| **Deprecated** \| **Index** \| **Help** | |  |
| PREV   NEXT | **FRAMES**    **NO FRAMES**     **All Classes** |  |


---


# Serialized Form


---

**Package** **org.testng**

**Class org.testng.SkipException extends java.lang.RuntimeException implements Serializable**

**serialVersionUID:** 4052142657885527260L

**Serialized Fields**

### m\_stackTrace

```
java.lang.StackTraceElement[] m_stackTrace
```

---

### m\_stackReduced

```
boolean m_stackReduced
```

**Class org.testng.SuiteRunner extends java.lang.Object implements Serializable**

**serialVersionUID:** 5284208932089503131L

**Serialized Fields**

### m\_suiteResults

```
java.util.Map<K,V> m_suiteResults
```

---

### m\_outputDir

```
java.lang.String m_outputDir
```

---

### m\_suite

```
XmlSuite m_suite
```

---

### m\_host

```
java.lang.String m_host
```

---

### m\_invokedMethodListeners

```
java.util.List<E> m_invokedMethodListeners
```

---

### m\_invokedMethods

```
java.util.List<E> m_invokedMethods
```

:   The list of all the methods invoked during this run

---

### m\_allTestMethods

```
java.util.List<E> m_allTestMethods
```

---

### m\_reporters

```
java.util.List<E> m_reporters
```

---

### m\_suiteState

```
SuiteRunState m_suiteState
```

---

### m\_attributes

```
IAttributes m_attributes
```

**Class org.testng.SuiteRunState extends java.lang.Object implements Serializable**

**serialVersionUID:** -2716934905049123874L

**Serialized Fields**

### m\_hasFailures

```
boolean m_hasFailures
```

**Class org.testng.TestException extends TestNGException implements Serializable**

**serialVersionUID:** -7946644025188038804L

**Class org.testng.TestNGException extends java.lang.RuntimeException implements Serializable**

**serialVersionUID:** -422675971506425913L

**Class org.testng.TestRunner extends java.lang.Object implements Serializable**

**serialVersionUID:** 4247820024988306670L

**Serialized Fields**

### m\_suite

```
ISuite m_suite
```

---

### m\_xmlTest

```
XmlTest m_xmlTest
```

---

### m\_testName

```
java.lang.String m_testName
```

---

### m\_allTestMethods

```
ITestNGMethod[] m_allTestMethods
```

:   All the test methods we found, associated with their respective classes.
    Note that these test methods might belong to different classes.
    We pick which ones to run at runtime.

---

### m\_startDate

```
java.util.Date m_startDate
```

---

### m\_endDate

```
java.util.Date m_endDate
```

---

### m\_outputDirectory

```
java.lang.String m_outputDirectory
```

:   Where the reports will be created.

---

### m\_xmlMethodSelector

```
org.testng.internal.XmlMethodSelector m_xmlMethodSelector
```

---

### m\_beforeSuiteMethods

```
ITestNGMethod[] m_beforeSuiteMethods
```

---

### m\_afterSuiteMethods

```
ITestNGMethod[] m_afterSuiteMethods
```

---

### m\_beforeXmlTestMethods

```
ITestNGMethod[] m_beforeXmlTestMethods
```

---

### m\_afterXmlTestMethods

```
ITestNGMethod[] m_afterXmlTestMethods
```

---

### m\_excludedMethods

```
java.util.List<E> m_excludedMethods
```

---

### m\_groupMethods

```
org.testng.internal.ConfigurationGroupMethods m_groupMethods
```

---

### m\_metaGroups

```
java.util.Map<K,V> m_metaGroups
```

---

### m\_passedTests

```
IResultMap m_passedTests
```

---

### m\_failedTests

```
IResultMap m_failedTests
```

---

### m\_failedButWithinSuccessPercentageTests

```
IResultMap m_failedButWithinSuccessPercentageTests
```

---

### m\_skippedTests

```
IResultMap m_skippedTests
```

---

### m\_runInfo

```
org.testng.internal.RunInfo m_runInfo
```

---

### m\_host

```
java.lang.String m_host
```

---

### m\_invokedMethods

```
java.util.List<E> m_invokedMethods
```

---

### m\_passedConfigurations

```
IResultMap m_passedConfigurations
```

---

### m\_skippedConfigurations

```
IResultMap m_skippedConfigurations
```

---

### m\_failedConfigurations

```
IResultMap m_failedConfigurations
```

---

### m\_attributes

```
IAttributes m_attributes
```

---

### m\_guiceModules

```
org.testng.collections.ListMultiMap<K,V> m_guiceModules
```

---

### m\_injectors

```
java.util.Map<K,V> m_injectors
```

**Class org.testng.TimeBombSkipException extends SkipException implements Serializable**

**serialVersionUID:** -8599821478834048537L

**Serialized Fields**

### m\_expireDate

```
java.util.Calendar m_expireDate
```

---

### m\_inFormat

```
java.text.DateFormat m_inFormat
```

---

### m\_outFormat

```
java.text.DateFormat m_outFormat
```

---

**Package** **org.testng.xml**

**Class org.testng.xml.XmlClass extends java.lang.Object implements Serializable**

**serialVersionUID:** 8885360896966149897L

**Serialized Fields**

### m\_includedMethods

```
java.util.List<E> m_includedMethods
```

---

### m\_excludedMethods

```
java.util.List<E> m_excludedMethods
```

---

### m\_name

```
java.lang.String m_name
```

---

### m\_class

```
java.lang.Class<T> m_class
```

---

### m\_index

```
int m_index
```

:   The index of this class in the  tag

---

### m\_loadClasses

```
boolean m_loadClasses
```

:   True if the classes need to be loaded

**Class org.testng.xml.XmlInclude extends java.lang.Object implements Serializable**

**serialVersionUID:** 1L

**Serialized Fields**

### m\_name

```
java.lang.String m_name
```

---

### m\_invocationNumbers

```
java.util.List<E> m_invocationNumbers
```

---

### m\_index

```
int m_index
```

---

### m\_description

```
java.lang.String m_description
```

---

### m\_parameters

```
java.util.Map<K,V> m_parameters
```

**Class org.testng.xml.XmlPackage extends java.lang.Object implements Serializable**

**serialVersionUID:** 1996341670354923204L

**Serialized Fields**

### m\_name

```
java.lang.String m_name
```

---

### m\_include

```
java.util.List<E> m_include
```

---

### m\_exclude

```
java.util.List<E> m_exclude
```

---

### m\_xmlClasses

```
java.util.List<E> m_xmlClasses
```

**Class org.testng.xml.XmlSuite extends java.lang.Object implements Serializable**

**serialVersionUID:** 4999962288272750226L

**Serialized Fields**

### m\_test

```
java.lang.String m_test
```

---

### m\_name

```
java.lang.String m_name
```

:   The suite name (defaults to DEFAULT\_SUITE\_NAME)

---

### m\_verbose

```
java.lang.Integer m_verbose
```

---

### m\_parallel

```
java.lang.String m_parallel
```

---

### m\_configFailurePolicy

```
java.lang.String m_configFailurePolicy
```

---

### m\_isJUnit

```
java.lang.Boolean m_isJUnit
```

---

### m\_isMixed

```
java.lang.Boolean m_isMixed
```

---

### m\_skipFailedInvocationCounts

```
java.lang.Boolean m_skipFailedInvocationCounts
```

---

### m\_threadCount

```
int m_threadCount
```

---

### m\_dataProviderThreadCount

```
int m_dataProviderThreadCount
```

---

### m\_groupByInstances

```
java.lang.Boolean m_groupByInstances
```

---

### m\_allowReturnValues

```
java.lang.Boolean m_allowReturnValues
```

---

### m\_xmlPackages

```
java.util.List<E> m_xmlPackages
```

:   The packages containing test classes.

---

### m\_expression

```
java.lang.String m_expression
```

:   BeanShell expression.

---

### m\_methodSelectors

```
java.util.List<E> m_methodSelectors
```

:   Suite level method selectors.

---

### m\_tests

```
java.util.List<E> m_tests
```

:   Tests in suite.

---

### m\_parameters

```
java.util.Map<K,V> m_parameters
```

:   Suite level parameters.

---

### m\_fileName

```
java.lang.String m_fileName
```

:   Name of the XML file

---

### m\_timeOut

```
java.lang.String m_timeOut
```

:   Time out for methods/tests

---

### m\_childSuites

```
java.util.List<E> m_childSuites
```

:   List of child XML suite specified using  tags

---

### m\_parentSuite

```
XmlSuite m_parentSuite
```

:   Parent XML Suite if this suite was specified in another suite using  tag

---

### m\_suiteFiles

```
java.util.List<E> m_suiteFiles
```

---

### m\_objectFactory

```
ITestObjectFactory m_objectFactory
```

---

### m\_listeners

```
java.util.List<E> m_listeners
```

---

### m\_preserveOrder

```
java.lang.String m_preserveOrder
```

---

### m\_includedGroups

```
java.util.List<E> m_includedGroups
```

---

### m\_excludedGroups

```
java.util.List<E> m_excludedGroups
```

**Class org.testng.xml.XmlTest extends java.lang.Object implements Serializable**

**serialVersionUID:** 6533504325942417606L

**Serialized Fields**

### m\_suite

```
XmlSuite m_suite
```

---

### m\_name

```
java.lang.String m_name
```

---

### m\_verbose

```
java.lang.Integer m_verbose
```

---

### m\_isJUnit

```
java.lang.Boolean m_isJUnit
```

---

### m\_threadCount

```
int m_threadCount
```

---

### m\_xmlClasses

```
java.util.List<E> m_xmlClasses
```

---

### m\_includedGroups

```
java.util.List<E> m_includedGroups
```

---

### m\_excludedGroups

```
java.util.List<E> m_excludedGroups
```

---

### m\_metaGroups

```
java.util.Map<K,V> m_metaGroups
```

---

### m\_parameters

```
java.util.Map<K,V> m_parameters
```

---

### m\_parallel

```
java.lang.String m_parallel
```

---

### m\_methodSelectors

```
java.util.List<E> m_methodSelectors
```

---

### m\_xmlPackages

```
java.util.List<E> m_xmlPackages
```

---

### m\_timeOut

```
java.lang.String m_timeOut
```

---

### m\_skipFailedInvocationCounts

```
java.lang.Boolean m_skipFailedInvocationCounts
```

---

### m\_failedInvocationNumbers

```
java.util.Map<K,V> m_failedInvocationNumbers
```

---

### m\_preserveOrder

```
java.lang.String m_preserveOrder
```

---

### m\_index

```
int m_index
```

---

### m\_groupByInstances

```
java.lang.Boolean m_groupByInstances
```

---

### m\_allowReturnValues

```
java.lang.Boolean m_allowReturnValues
```

---


|  |  |  |
| --- | --- | --- |
| **Overview** \| Package \| Class \| **Tree** \| **Deprecated** \| **Index** \| **Help** | |  |
| PREV   NEXT | **FRAMES**    **NO FRAMES**     **All Classes** |  |


---
